# Supplementary material for: Economic burden of sickle cell disease in Brazil
Source: PLoS One. 2022 Jun 16;17(6):e0269703. doi: 10.1371/journal.pone.0269703 (PMC9202914; doi:10.1371/journal.pone.0269703)
Supplement: S1 File — (DOCX) [file pone.0269703.s001.docx]

**Supplementary Material**

| **Parameters** | **Base value** | **Lower limit** | **Higher Limit** | **Reference** |
| --- | --- | --- | --- | --- |
| SCD prevalence | 0.00024 | 0.00012 | 0.00048 | PCDT |
| Annual income | 28776.00 | 23020.80 | 34531.20 | ±20% |
| SCD mortality rate | 0.01 | 0.01 | 0.01 | Lobo, 2018 |
| SCD base utility | 0.73 | 0.58 | 0.88 | ±20% |
| VOC annualized rate | 5.30 | 0.00 | 12.10 | Osunkwo, 2020 |
| Acute complications annualized event rate: Vaso-occlusion | 5.30 | 0.00 | 12.10 | ±20% |
| Annual cost - Standard of care (Adults) | 7123.37 | 5698.70 | 8548.04 | ±20% |
| Annual cost - Standard of care (Children) | 3830.39 | 3064.31 | 4596.47 | Osunkwo, 2020 |
| Annual cost - Acute complications (Children) | 2730.38 | 2184.30 | 3276.45 | ±20% |
| % of adults | 0.44 | 0.41 | 0.51 | Carneiro-Proietti, 2018 |
| Annual cost - Acute complications (Adults) | 2311.25 | 1849.00 | 2773.50 | ±20% |
| Acute complications incidence (Children): Vaso-occlusion | 0.60 | 0.51 | 0.68 | Belini Junior |
| Chronic complications incidence (Adults): chronic kidney disease (failure) | 0.04 | 0.03 | 0.05 | Loureiro |
| Acute complications incidence (Adults): Vaso-occlusion | 0.75 | 0.67 | 0.83 | ±20% |
| Acute complications incidence (Children): Hand-Foot syndrome | 0.25 | 0.20 | 0.30 | ±20% |
| Acute complications annualized event rate: Hand-Foot syndrome | 5.30 | 4.24 | 6.36 | ±20% |
| Acute complications annualized event rate: Infections | 0.12 | 0.09 | 0.14 | ±20% |
| Chronic complications incidence (Adults): Cardiac complications | 0.16 | 0.09 | 0.22 | ±20% |
| Acute complications incidence (Children): Infections | 0.50 | 0.40 | 0.60 | ±20% |
| Chronic complications incidence (Adults): Pulmonary hypertension | 0.10 | 0.05 | 0.15 | ±20% |
| Acute complications annualized event rate: Acute thoracic syndrome | 0.11 | 0.09 | 0.13 | ±20% |
| Annual cost - HSCT (Adults) | 236.45 | 189.16 | 283.73 | Bukar, 2019 |
| Chronic complications incidence (Adults): Osteoporosis | 0.25 | 0.17 | 0.32 | Belini Junior |
| Annual cost - HSCT (Children) | 153.69 | 122.95 | 184.43 | Belini Junior |
| Chronic complications incidence (Adults): chronic kidney disease (without failure) | 0.39 | 0.30 | 0.47 | Baldanzi |
| Chronic complications incidence (Children): Cardiac complications | 0.03 | 0.00 | 0.05 | ±20% |
| Acute complications incidence (Adults): Infections | 0.32 | 0.26 | 0.38 | SWAY BR |
| Chronic complications incidence (Adults): Retinopathy | 0.37 | 0.29 | 0.46 | Lima, 2006 |
| Chronic complications incidence (Adults): Chronic liver disease | 0.17 | 0.10 | 0.24 | ±20% |
| Acute complications incidence (Children): Acute thoracic syndrome | 0.55 | 0.46 | 0.64 | ±20% |
| Chronic complications incidence (Children): Chronic liver disease | 0.08 | 0.03 | 0.13 | Fonseca, 2012 |
| Chronic complications incidence (Adults): Leg ulcers | 0.18 | 0.11 | 0.25 | ±20% |
| Chronic complications incidence (Children): Chronic kidney disease (without failure) | 0.20 | 0.16 | 0.24 | ±20% |
| Calculated VOC Decrement (per Event) | 0.01 | 0.01 | 0.01 | Rezende, 2009 |
| Acute complications incidence (Children): Splenic sequestration | 0.35 | 0.27 | 0.43 | ±20% |
| Days Hospitalized per VOC | 5.59 | 4.47 | 6.71 | ±20% |
| Acute complications annualized event rate: Splenic sequestration | 0.09 | 0.07 | 0.11 | ±20% |
| Chronic complications incidence (Children): Calculous chronic cholecystitis | 0.19 | 0.12 | 0.26 | ±20% |
| Chronic complications incidence (Adults): Calculous chronic cholecystitis | 0.62 | 0.53 | 0.71 | ±20% |
| Acute complications duration (days): Vaso-occlusion | 14.59 | 11.67 | 17.51 | SWAY BR |
| Acute complications disability weights: Vaso-occlusion | 0.01 | 0.01 | 0.01 | ±20% |
| Utility Decrement During VOC | 0.36 | 0.29 | 0.43 | Carneiro-Proietti, 2018 |
| Chronic complications incidence (Children): Osteoporosis | 0.10 | 0.08 | 0.12 | ±20% |
| Acute complications incidence (Adults): Acute thoracic syndrome | 0.30 | 0.24 | 0.36 | SWAY BR |
| Days Post-Hospitalization | 7.00 | 5.60 | 8.40 | ±20% |
| Acute complications incidence (Adults): Stroke | 0.15 | 0.08 | 0.21 | SWAY BR |
| Chronic complications incidence (Children): Recurrent priapism | 0.07 | 0.02 | 0.11 | ±20% |
| Acute complications annualized event rate: Stroke | 0.12 | 0.10 | 0.14 | ±20% |
| Acute complications annualized event rate: Cholelithiasis | 0.09 | 0.08 | 0.11 | Belini Junior |
| Chronic complications incidence (Children): Chronic kidney disease (failure) | 0.00 | 0.00 | 0.00 | ±20% |
| Acute complications incidence (Adults): Deep venous thrombosis | 0.10 | 0.05 | 0.15 | Belini Junior |
| Days of Pain Prior to Hospitalization (per VOC) | 2.00 | 1.60 | 2.40 | ±20% |
| Acute complications incidence (Children): Cholelithiasis | 0.27 | 0.19 | 0.35 | Carneiro-Proietti, 2018 |
| Chronic complications incidence (Adults): Recurrent priapism | 0.15 | 0.12 | 0.18 | Martins, 2017 |
| Acute complications incidence (Adults): Cholelithiasis | 0.35 | 0.27 | 0.44 | 20% |
| Acute complications incidence (Children): Stroke | 0.10 | 0.08 | 0.12 | 20% |
| Chronic complications incidence (Children): Leg ulcers | 0.01 | 0.00 | 0.02 | 20% |
| Utility Decrement for Post-VOC Period | 0.10 | 0.08 | 0.12 | 20% |
| Chronic complications incidence (Children): Retinopathy | 0.05 | 0.04 | 0.06 | 20% |
| Acute complications duration (days): Acute thoracic syndrome | 7.00 | 5.60 | 8.40 | 20% |
| Acute complications disability weights: Acute thoracic syndrome | 0.33 | 0.26 | 0.40 | 20% |
| Acute complications duration (days): Hand-Foot syndrome | 15.00 | 12.00 | 18.00 | Carneiro-Proietti, 2018 |
| Acute complications disability weights: Hand-Foot syndrome | 0.01 | 0.01 | 0.01 | 20% |
| Acute complications annualized event rate: Priapism | 0.14 | 0.11 | 0.17 | 20% |
| Acute complications incidence (Adults): Osteonecrosis | 0.14 | 0.07 | 0.20 | Fonsea, 2012 |
| Acute complications incidence (Adults): Priapism | 0.26 | 0.18 | 0.34 | Belini Junior |
| Acute complications incidence (Children): Priapism | 0.13 | 0.07 | 0.19 | 20% |
| Acute complications annualized event rate: Deep venous thrombosis | 0.12 | 0.10 | 0.14 | 20% |
| Chronic complications incidence (Children): Pulmonary hypertension | 0.01 | 0.01 | 0.01 | 20% |
| Acute complications duration (days): Infections | 5.00 | 4.00 | 6.00 | Belini Junior |
| Acute complications disability weights: Infections | 0.21 | 0.17 | 0.25 | Belini Junior |
| Acute complications annualized event rate: Osteonecrosis | 0.12 | 0.09 | 0.14 | 20% |
| Acute complications incidence (Children): Osteonecrosis | 0.02 | 0.00 | 0.04 | 20% |
| Acute complications incidence (Adults): Splenic sequestration | 0.02 | 0.02 | 0.02 | 20% |
| Acute complications duration (days): Splenic sequestration | 6.00 | 4.80 | 7.20 | 20% |
| Acute complications disability weights: Splenic sequestration | 0.19 | 0.16 | 0.23 | 20% |
| Acute complications annualized event rate: Liver sequestration | 0.11 | 0.09 | 0.13 | 20% |
| Acute complications incidence (Children): Liver sequestration | 0.02 | 0.02 | 0.02 | Carneiro-Proietti, 2018 |
| Acute complications incidence (Children): Deep venous thrombosis | 0.02 | 0.02 | 0.02 | 20% |
| Acute complications duration (days): Cholelithiasis | 5.00 | 4.00 | 6.00 | 20% |
| Acute complications disability weights: Cholelithiasis | 0.11 | 0.09 | 0.14 | 20% |
| Acute complications duration (days): Deep venous thrombosis | 10.00 | 8.00 | 12.00 | 20% |
| Acute complications disability weights: Deep venous thrombosis | 0.18 | 0.14 | 0.21 | 20% |
| Acute complications duration (days): Osteonecrosis | 4.00 | 3.20 | 4.80 | 20% |
| Acute complications disability weights: Osteonecrosis | 0.26 | 0.21 | 0.31 | 20% |
| Acute complications duration (days): Stroke | 9.00 | 7.20 | 10.80 | 20% |
| Acute complications disability weights: Stroke | 0.08 | 0.06 | 0.09 | Belini Junior |
| Acute complications duration (days): Liver sequestration | 6.00 | 4.80 | 7.20 | 20% |
| Acute complications disability weights: Liver sequestration | 0.18 | 0.14 | 0.21 | 20% |
| Acute complications incidence (Adults): Liver sequestration | 0.00 | 0.00 | 0.00 | 20% |
| Annual cost - Chronic complications (Adults) | 2984.89 | 2387.91 | 3581.87 | 20% |
| Annual cost - Chronic complications (Children) | 452.48 | 361.99 | 542.98 | 20% |
| Acute complications incidence (Adults): Hand-Foot syndrome | 0.00 | 0.00 | 0.00 | 20% |
| Acute complications duration (days): Priapism | 5.00 | 4.00 | 6.00 | 20% |
| Acute complications disability weights: Priapism | 0.00 | 0.00 | 0.00 | 20% |
| Mortality HR (vs. general population) | 4.70 | 3.76 | 5.64 | 20% |
