# Supplementary material for: Economic burden of sickle cell disease in Brazil
Source: PLoS One. 2022 Jun 16;17(6):e0269703. doi: 10.1371/journal.pone.0269703 (PMC9202914; doi:10.1371/journal.pone.0269703)
Supplement: Attachment — Submitted filename: Response to Reviewers.docx [file pone.0269703.s002.docx]

Dear reviewers,

We would like to thank you for reviewing the article and to state that suggestions were considered in the manuscript revised version. Below we provide a point-by-point response to all proposed modifications:

Reviewer #1:

The authors use publicly available data to determine the economic burden to the society of providing medical care for people living with SCD (PLWSCD). They include indirect costs, an important component of total economic cost.

ABSTRACT

The English can be improved. For example, “Sickle cell disease (SCD) may cause several impacts to patients and the whole society.”

- Answer: The article was fully reviewed to improve English.

The use of the BRL is difficult for the international audience. Consider using a commonly understood currency equivalent such as the USD or GBP, or at least stating whet he exchange rate was in the captioned year.

- Answer: The currency used was changed to USD.

INTRODUCTION
The English can be improved. While it is difficult to give specific examples, the manuscript would benefit from thorough review by a proofreader. Some examples include “composed heterozygous disease”. “Furthermore, a mortality rate of 1.12/100,000 habitants is estimated”, “Only hematopoietic stem cell transplantation has a curative intention, however, for few patients.”, “disparities on SCD patients’ care”, “It was possible to notice that mortality rates are still higher in young ages, such as children.”.

- Answer: The article was fully reviewed to improve English.

The mortality quoted comes from a specialist hospital “Furthermore, a mortality rate of 1.12/100,000 habitants is estimated [6]”. It has been shown that this is a biased (more severe) population and that ideally cohorts assembled at newborn screening are the best approach. Given that “In Brazilian context, direct non-medical costs, such as transport for medical appointments, may impose important burden since disease reference sites are located in few places.”, PLWSCD who are well may not make the trip. This limitation should be acknowledged.

- Answer: Thank you for the suggestion. Mortality rate was set as 1.12% per year in patients with SCD, which results in a mortality rate of 2.69/1,000,000 habitants. The mortality rate of Santo (2020) ranges between 2.65 and 2.7 per million habitants. The reference was updated to Santo (2020) as it’s a more recent one (Page 9; Lines 7-8). However, we also included the potential limitation in discussion section (Page 18; Line 33. Page 19; Lines 1-3).

The authors say that “Considering the disease burden, many interventions such as neonatal screening, bacterial prophylaxis and a comprehensive healthcare management have been established [2]”, quoting an international commentary and not speaking to how often these interventions are applied in Brazil.

- Answer: A statement about Brazilian scenario on such issues was included (Page 5; Lines 8-10).

Similarly, the cost of care quoted was from Florida. “Kauf et al. (2009) reported an average total cost of care per patient-month of USD1,946, however only direct medical costs were included [16].”

- Answer: A new study, published in 2022, reporting the Brazilian scenario was included. However, a statement highlighting the absence of cost of illness studies conducted in Brazil was also included in the introduction (Page 5; Lines 23-26 XX).

The population of Brazil is approximately 212.6 million. The upper limit of the estimated SCD population is 100,000. This gives a maximum estimate of prevalence of 0.5% of the total population having SCD. The fact that people living with SCD (PLSCD) have related deaths and morbidity does not clearly show that “As a consequence, SCD patients have greater morbidity and mortality which could impact the whole society”.

- Answer: Indeed, SCD morbidity and mortality may not have an impact to the whole Brazilian society. The statement was excluded, and the paragraph was rewritten to highlight the importance of the study on providing information to support health policy making decisions in the country. In addition, a statement about Brazilian healthcare system structure was included, justifying the impact for the whole society (Page 5; Lines 2-6).

There should be a reference at the end of this critical sentence “The analysis followed the methodology proposed by Larg & Moss (2011) for a cost-of-illness evaluation”.

- Answer: The reference was included (Page 6, Line 16).

Agree that reference is missing “Direct medical costs were estimated using a bottom-up strategy with the identification of the main health states related to SCD (Error! Reference source not found.)”.

- Answer: Methods section was fully rewritten, and this reference was excluded.

There are references published before October 2019 which have been omitted. Arduini (2017) looks at several studies regarding SCD mortality in Brazil including one by Ramos (2015) from which he quotes an estimate of a mortality coefficient of 0.54 per 100,000 individuals.

- Answer: Thank you for the suggestion. Arduini et al (2017) was referenced and data was included in introduction (Page 5; Lines 12-16).

Figures 1 and 2 are unhelpful. It is not clear what they are saying.

- Answer: Figures 1 and 2 were excluded, as proposed.

METHODS
Given that it is now 2022, the halting of the literature review more than two years ago is unacceptable. There have been significant publications since then which must be mentioned in discussing how use of the different estimates may have affected the final outcomes. “Literature review was performed until October 2019”. Santo et al (2020) estimate annual mortality rates at approximately 2.65 and 2.7 per million for men and women respectively. This is very different from the 1.1% used here and would impact estimates significantly. Sensitivity analyses for these possibilities would be necessary. The YLL would be significantly lower using these estimates.

- Answer: Mortality rate was set as 1.12% per year in patients with SCD, which results in a mortality rate of 2.69/1,000,000 habitants. The mortality rate of Santo (2020) ranges between 2.65 and 2.7 per million habitants. The reference was updated to Santo (2020) as it’s a more recent one (Page 9; Lines 7-8).

What does this sentence mean? “Disability attributed to chronic complications was incorporated from the sickle cell anemia DW”.

- Answer: The sentence was removed, and the section was totally rewritten for improved clarity.

RESULTS
It is not clear where the following sentence comes from. Is it a result arising from this research? “A prevalence of 24.0 cases per 100,000 inhabitants was considered, resulting in approximately 50,000 patients with the disease in 2018, segmented by age according to the IBGE population projection [24].”

- Answer: Prevalence was derived from medical opinion. This was clarified in the text (Page 9; Line 5).

Similarly, is the calculation all done on the opinion of the medical expert? Most of the calculated costs come from indirect costs which are based on these premises.
“Considering the scarcity of information and that disease prevalence in Brazil varies in the available literature, data was defined as an assumption proposed by ME. A disease specific mortality rate of 1.12% was assumed, resulting in 560 patients lost to the disease in the same year [6]. The quoted study is not the only publication on SCD mortality in Brazil.

- Answer: Disease prevalence was based on ME opinion. Mortality rate was updated to use Santo (2020), which doesn’t differ significantly from the reference above.

Table1. For acute complications, please clarify whether the presented %s are for the captioned year. Did 26% of all PLWSCD, and therefore approximately 52% of all men have priapism annually? Did 35.4% of PLWSCD have cholelithiasis annually;14.4% stroke, 32% infections (not including ACS)?

- Answer: The acute complications presented was evaluate for the captioned year and all of them were capture by the medical expert opinion considering the literature and their on assumptions from their reference centers.

The opinions of medical experts can be influenced by their type of practice. Those in secondary or tertiary care may not see the asymptomatic persons. An example is proliferative sickle retinopathy. The reference (17). The estimate is taken from a single, cross-sectional study and ME. The estimate is much higher than that in a cohort study.

- Answer: Indeed, opinions of medical experts can be influenced by their practice. However, such strategy was used to deal with the lack of information in the literature.

Is Figure 3 Are the deaths shown from SCD or the general population?

- Answer: Results are from SCD population. Figure legend was fixed to provide a clear information (Page 9; Line 15).

Are the YLL based on those who died only in the year under review?

- Answer: No. YLL was calculated based on the life expectancy method. Thus, productivity lost due to early death was accounted (ie: if someone dies at age 21 and life expectancy is 75 years, 54 years of productivity were lost).

More details are needed on how disability was assigned. I do not understand how the DW were calculated. The DW do not make intuitive sense to me. Stroke has DW of 0.08, but this acute episode has chronic disability associated. ACS which usually has no disability after the episode has a DW of 0.33.

- Answer: DW were reviewed, and the appropriate reference added to Table 1. DW assignment method was defined in Methods section (Page 7; Lines 3-11).

How is the indirect cost calculated? In the last part of Table 4 (see below), the direct cost is $479,105,564. Is the DALY not the source of the indirect costs? By what calculation is the DALY 22,755 transformed to the indirect cost 654,790,110

- Answer: This is better clarified by newly added equations at the Methods section (Page 7).

Figure 4. This needs a proper title stating what Tornado diagram depicts.

- Answer: Figure legend was fixed to provide a clearer information (Page 15; Line 14).

What does SCD prevalence represent in terms of costs in the Tornado diagram?

- Answer: Parameter limits were added to the Tornado diagram, as well as the spread of the results when each parameter is changed from base case.

Discussion
The high death rate among children is surprising, given that there is newborn screening, vaccines and prophylactic antibiotics. In another developing country, Jamaica, the mortality in children has fallen dramatically with these few, inexpensive interventions. More explanation is needed, such as the causes of death, given that these interventions are available in Brazil and hydroxyurea use (50%,29%) is relatively high for non non-developed country.

- Answer: You are correct, differences in mortality were highlighted. We have included other references that contributes to explain such differences (Page 16; Lines 16-24).

Are there publications from Brazil about economic burden of other diseases? This would aid in putting these results into context.

- Answer: References related to economic burden of hematologic diseases were included as suggested (Page 16; Lines 6-8).

The other estimates of mortality rates should be discussed.

- Answer: The reference used to determine mortality rate was changed, following reviewers’ suggestion. In addition, discussion on mortality rates was improved (Page 16; Lines 9-24).

Reviewer #2:

In this article, Carolina Bueno and colleagues estimate the monetary burden of Sickle Cell Disease (SCD) in Brazil and its effect and burden on the overall Brazilian Health care. The authors build a model using a one-year time horizon and considering direct medical costs and also indirect costs related to morbidity and mortality. The study concluded that burden for SCD care is at 1.6 billion Brazilian reals (approximately $300 million) pear year. The presented data also point to early childhood mortality, not seen in US, for example, and vaso-occlusive crises as the most frequent complication of SCD. Overall this is an interesting study, which is the first to estimate the burden of SCD in Brazil. The study provides a wealth of useful data and will be helpful for future public policy making decisions in Brazil. One major drawback is that the model is not well described (see detailed comments below). Also the authors need to fix numerous minor errors and provide additional citations and expand the discussion.

MAJOR CRITIQUES:

1. Please provide more clear description of the model.

- Answer: Methods section was fully rewritten to provide more clear descriptions.

2. Please, provide more information about direct medical cost estimation. What is bottom-up strategy? Micro-costing method? Are there reference papers describing this methodology and strategy?

- Answer: Methods section was fully rewritten to provide more clear descriptions.

3. Figure 3 needs to have axis labels. It is unclear if this graph displays the estimated number of deaths per age group. Were these values per year or for the whole study, since data analysis was performed from 2008 to 2018? Please, clarify this in the text and also in the figure legend.

- Answer: The figure was fixed, and information included in the text (Page 9; Lines 5-6).

4. Authors should be more specific regarding which criteria and how medical experts validated complicated estimates.

- Answer: Methods section was fully rewritten to provide more clear descriptions.

5. Discussion, paragraph 9: you can expand the discussion and add references regarding deterministic sensitivity analysis and the influence of SCD prevalence, annual income and SCD base utility in disease burden.

- Answer: Discussion was expanded as suggested (Page 18; Lines 21-28).

MINOR CHANGES:

1. Page numbers need to be added.

- Answer: Page numbers were added.

2. Introduction, lane 5: “haemoglobin SS” – please change to “hemoglobin SS”. Also change throughout the manuscript.

- Answer: The term “haemoglobin” was changed throughout the whole document.

3. Introduction, lane 7: “it is globally observed due to population migration.” – Slave trade is not exactly population migration, I suggest rephrasing it to clarify.

- Answer: The term was excluded from the text.

4. Introduction, lane 8: you may consider abbreviating sickle cell anemia as SCA.

- Answer: Sickle cell anemia was abbreviated as SCA, following the suggestion.

5. Page 11, second paragraph: most of the available studies report only costs among hospitalized patients. However, Shah and coworkers (2020) reported the impact of vaso-occlusive crises in disease burden in non-hospitalized patients. Saraf and coworkers (2020) also demonstrated the cost impact of disease in a more specific SCD population. These manuscripts might be interesting to add to the discussion.

- Answer: Thank you for the suggestion. The study conducted by Shah and coworkers was included (Page 5; Lines 21-22). However, considering that Saraf and colleagues assess a specific population, transplanted and non-transplanted adults with sickle cell disease, the reference was not included.

6. Tables 1, 2 and 3: References do not specify if they refer to children or adults. Please, clarify.

- Answer: Tables were fixed to provide a clear information about references used.

7. Table 3, please identify the acronym “SWAY”.

- Answer: The acronym was included as proposed.

8. Page 12: “the main health states related to SCD (Error! Reference source not found.)” – please correct references.

- Answer: References were fixed throughout the whole text.

9. Page 14, lane 6 “ data was defined” - “ data were defined.

- Answer: Wording was fixed.

10. Page 20, lane 25 “ estimated total DALY lost in 2018” should be “estimated total DALY loss in 2018”

- Answer: Wording was fixed.
